# A novel secretion and online-cleavage strategy for production of cecropin A in *Escherichia coli*

**DOI:** 10.1038/s41598-017-07411-5

**Published:** 2017-08-04

**Authors:** Meng Wang, Minhua Huang, Junjie Zhang, Yi Ma, Shan Li, Jufang Wang

**Affiliations:** 1 School of Bioscience and Bioengineering, South China University of Technology, Guangzhou, 510006 China; 2 Guangdong Key Laboratory of Fermentation and Enzyme Engineering, School of Bioscience and Bioengineering, South China University of Technology, Guangzhou, 510006 China

## Abstract

Antimicrobial peptides, promising antibiotic candidates, are attracting increasing research attention. Current methods for production of antimicrobial peptides are chemical synthesis, intracellular fusion expression, or direct separation and purification from natural sources. However, all these methods are costly, operation-complicated and low efficiency. Here, we report a new strategy for extracellular secretion and online-cleavage of antimicrobial peptides on the surface of *Escherichia coli*, which is cost-effective, simple and does not require complex procedures like cell disruption and protein purification. Analysis by transmission electron microscopy and semi-denaturing detergent agarose gel electrophoresis indicated that fusion proteins contain cecropin A peptides can successfully be secreted and form extracellular amyloid aggregates at the surface of *Escherichia coli* on the basis of *E. coli* curli secretion system and amyloid characteristics of sup35NM. These amyloid aggregates can be easily collected by simple centrifugation and high-purity cecropin A peptide with the same antimicrobial activity as commercial peptide by chemical synthesis was released by efficient self-cleavage of *Mxe* GyrA intein. Here, we established a novel expression strategy for the production of antimicrobial peptides, which dramatically reduces the cost and simplifies purification procedures and gives new insights into producing antimicrobial and other commercially-viable peptides.

## Introduction

Because of their potent, fast, long-lasting activity against a broad range of microorganisms and lack of bacterial resistance, antimicrobial peptides (AMPs) have received increasing attention^1^. Over recent years, many antibacterial peptides have been produced by direct separation and purification from natural sources^4^, or by chemical synthesis^2, 3^. However, the low efficiency, low yield and costly methods hinder the large-scale production of AMPs. Several AMPs derived from animals or insects have been produced by recombinant expression in prokaryotic systems, and the most frequently used is *Escherichia coli* ^5–8^. However, almost all AMPs produced in *E. coli* are intracellular, so cell disruption and expensive purification procedures are required. Moreover, problems associated with proteolytic degradation of peptides during expression and antimicrobial activity toward the host system mean this is not an ideal choice for production of AMPs. Research and development of novel secretory expression systems may be worthwhile.

A variety of secretory expression systems have been developed. The antibacterial peptide apidaecin was produced in a *Streptomyces* secretory expression system^9^; AMP PR39 was successfully expressed and secreted via a maltose-inducible vector in *Bacillus subtilis* ^10^; five recombinant AMPs using the catalytic domain of a cellulase as fusion partner were secreted from *E. coli* ^11^. These attempts to produce AMPs using different secretory systems have shown that the strategy of secretory expression is practical and feasible. However, current secretory strategies still require high-cost purification procedures (e.g., metal chelate affinity chromatography) from secretions and the toxicity of AMPs to host exists to some extent. The strategy of fusion proteins with low toxicity to host cells is an urgent need.

Recently, a new strategy for the secretion of peptides via the *E. coli*-based curli secretion system was reported^12^. Curli, functional extracellular amyloid fibers assembled by many Enterobacteriaceae, are involved in adhesion, cell aggregation, and biofilm formation^13, 14^. Curli biogenesis is precisely controlled by the divergently transcribed *csgBA* and *csgDEFG* operons^15^. Briefly, CsgA monomers are secreted to the cell surface and interact with CsgB, which nucleates the polymerization of the secreted CsgA subunits, and then self-assemble into curli fibers^16^. CsgD, a positive transcriptional regulator of the *csgBA* operon, regulates the expression of *csgA* and *csgB* according to environmental stress (nutrients, oxygen tension, temperature)^17^. CsgE and CsgF are proteins facilitating secretion of CsgA by interacting with CsgG at the outer membrane (OM); CsgG, an OM lipoprotein, plays a key role in secreting CsgA and CsgB^18–20^.

**Figure 1:**
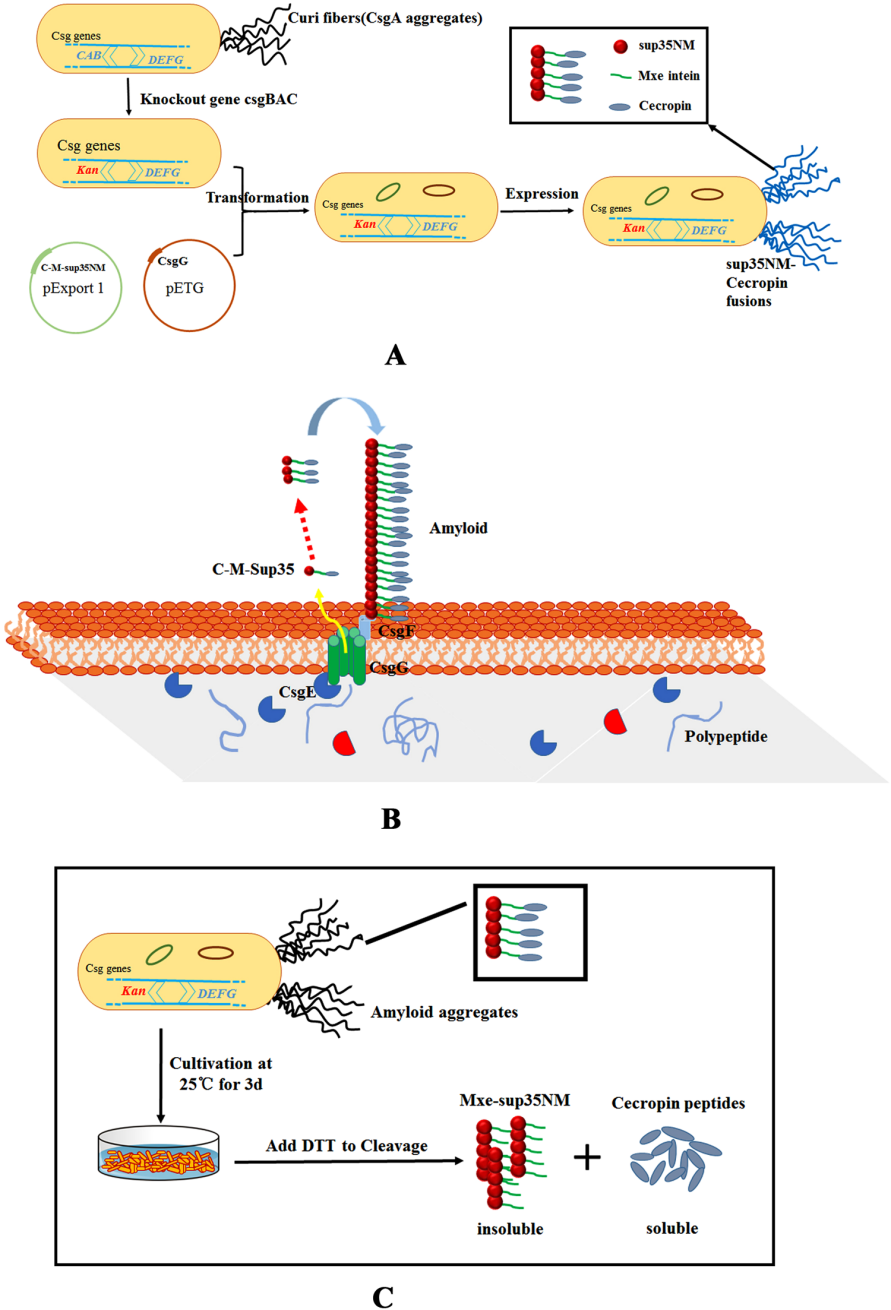
The technical roadmap for extracellular secretion and online-cleavage of antimicrobial peptides on the surface of *Escherichia coli*. (**A**) Strategy for generating extracellular fiber-like aggregates based on the *E. coli* curli system. A curli-deficient mutant of *E. coli* BL21 (DE3) was constructed by deleting *csgBAC* and replacing them with a kanamycin resistance gene. pExport 1, encoding a C-M-sup35NM fusion protein, and pETG, directing the overexpression of CsgG, were introduced into strain BL21 (DE3) ∆*csgBAC*. The recombinant bacteria were then cultured on YESCA plates supplemented with appropriate antibiotics and inducers at 25 °C for 3 d to produce fiber-like aggregates (blue fibers). The black box shows the composition of the fiber-like aggregates. (**B**) Model of secretion and assembly principle CsgG forms an ungated peptide diffusion channel in the cell outer membrane (OM). C-M-sup35NM fusions firstly pass through the inner membrane (IM) into the periplasm via the Sec-pathway, and are then secreted to the cell surface of BL21 (DE3) ∆*csgBAC* in a CsgG- and CsgE-dependent manner. The secreted C-M-sup35NM fusions tend to form fiber-like aggregates because of the amyloid of sup35NM. CsgF associates with the OM and is required for adhesion of the C-M-sup35NM aggregates. (**C**) Schematic diagram of cleavage experiment mediated by *Mxe* GyrA BL21 (DE3) ∆*csgBAC* harboring pExport1 and pETG were cultivated on YESCA plates supplemented with the appropriate antibiotics (100 µg/ml ampicillin; 50 µg/ml kanamycin; 25 µg/ml chloramphenicol) and inducers (0.05% [w/v] L-arabinose, 1 mM IPTG) at 25 °C for 3 d. Subsequently, cells were harvested and resuspended in buffer S1 (20 mM Tris-HCl, 500 mM NaCl, 1 mM disodium edetate (EDTA), pH 8.5) to 10 OD_600_ culture/ml, followed by centrifugation at 7500 *g* for 15 min at 4 °C. The precipitates were washed twice with buffer S1, and resuspended in the same volume of buffer S2 (20 mM Tris-HCl, 500 mM NaCl, 1 mM EDTA and 40 mM dithiothreitol (DTT), pH 8.5) for cleavage at 4 °C for 12 or 24 h. Cecropin A peptide (soluble) was released and collected by centrifugation at 11000 rpm for 30 min. The M-sup35 fusion (insoluble) formed part of the pellet. The black box shows the composition of the fiber-like aggregates.

In previous studies, sup35NM, a yeast prion protein, was secreted from *E. coli* by the curli export system and formed extracellular amyloid fibers at the cell surface^21^. According to some reports in recent years, *Mxe* GyrA, a mini intein which can be cleaved at its N-terminal by the addition of a thiol nucleophile such as DTT, is widely applied for fusion protein expression of small or toxic proteins because of its high cleavage efficiency^22, 23^. Based on the results of that study, a cecropin A-*Mxe* GyrA-sup35NM fusion protein (C-M-sup35NM) was constructed here to achieve secretion and aggregation at the cell surface. Figure 1 shows the technical roadmap. A curli-deficient mutant was constructed as the expression host, and then two compatible plasmids, encoding CsgG and the C-M-sup35NM fusion protein respectively, were transformed into the mutant to achieve secretion (Fig. 1A). As shown in Fig. 1B, fusions with the N-terminal 42 amino acids of premature CsgA (including the CsgA Sec dependent signal sequence and the CsgG targeting sequence) were first translocated across the inner membrane into the periplasm through the general Sec translocation system, and then secreted to the cell surface with the help of curli-specific CsgG^24, 25^. Because of the amyloid characteristics of sup35NM, secreted C-M-sup35NM fusions tended to aggregate and form amyloid fibers. Moreover, the self-cleaving peptide *Mxe* GyrA (which could be self-cleaved at its N-terminus by the addition of thiol agent) was also inserted to release the cecropin A peptide on the addition of a thiol agent such as dithiothreitol (DTT) (Fig. 1C). Eventually, high purity of cecropin A peptide was obtained by a simple ultrafiltration with 3 kDa ultrafiltration tube at 4500 *g*.

In this study, a novel method of secretion expression and online-cleavage was developed. Small proteins such as AMPs and cell growth factors can be successfully secreted and easily collected by one-step cleavage at the cell surface. Tedious cell disruption and expensive purification steps are no longer needed, which provides a new way of research into small proteins and a novel platform for large-scale production of AMPs.

## Results and Discussion

### Construct design, cloning and identification of mutant strain *E. coli* BL21 (DE3) ∆*csgBAC*

**Figure 2:**
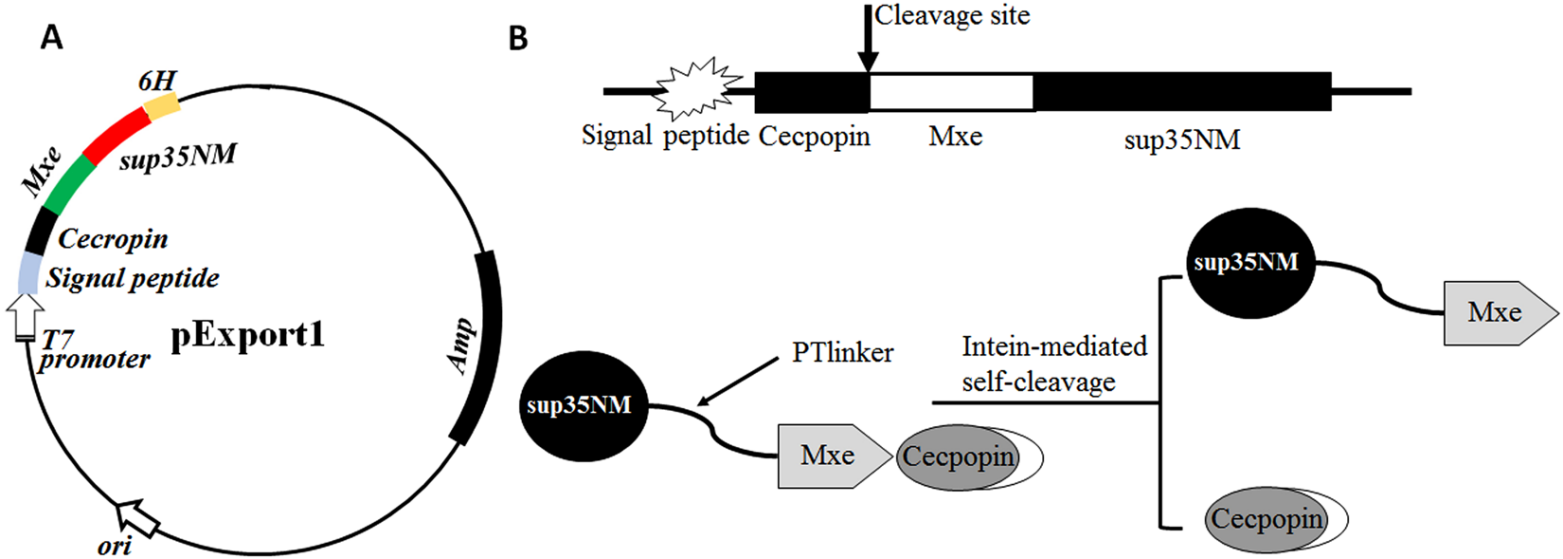
Construction of C-M-sup35NM fusion proteins. (**A**) Plasmid map. (**B**) Schematic design of fusion sequences. Residues from the N-terminus of CsgA were used as a signal peptide.

In our study, a fusion construction was composed of three elements: an antibacterial peptide (cecropin A), *Mxe* GyrA^26^, and prion sup35NM^27^. The general scheme for fusion construction is shown in Fig. 2. Specifically, the first 42 residues of CsgA formed a signal peptide at the N-terminus, the *Mxe* GyrA intein was fused to the C-terminus of cecropin A, and a nonpathogenic yeast prion-like protein sup35NM was incorporated at the C-terminus of the fusion (Fig. 2A). *Mxe* GyrA intein can be self-cleaved at its N-terminus by the addition of a thiol agent such as DTT, and cecropin A will thus be released from the fusion by incubation with DTT (Fig. 2B).

**Figure 3:**
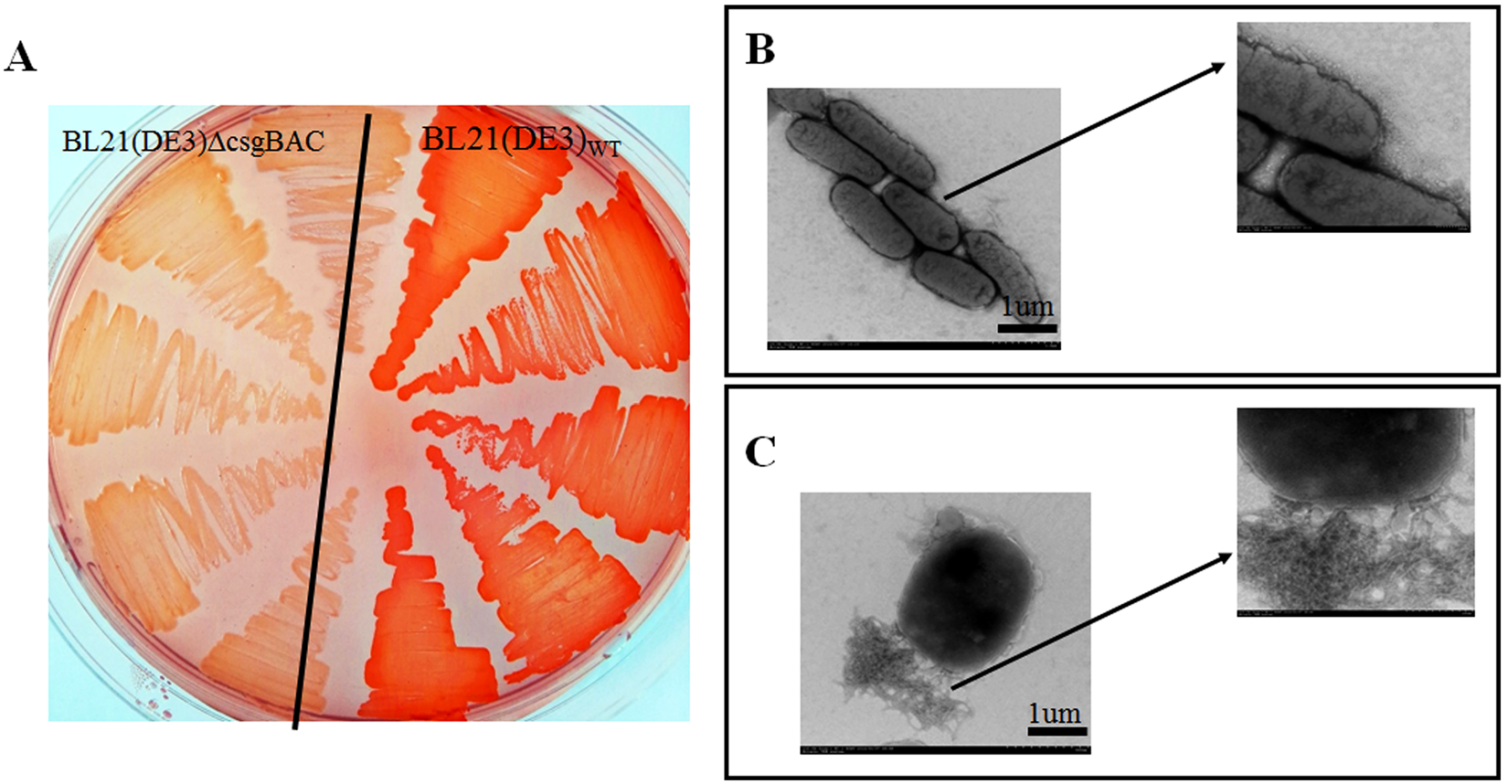
Phenotype of BL21 (DE3)_WT_ and BL21 (DE3) ∆*csgBAC* after growth on a YESCA plate. (**A**) BL21 (DE3)_WT_ and BL21 (DE3) ∆*csgBAC* were cultured on YESCA-Congo red indicator plates at 25 °C for 3 d. Red staining indicates amyloid production. BL21 (DE3) ∆*csgBAC* appeared white, which indicates that this strain may have lost the ability to produce curli fibers. Transmission electron microscopy (TEM) analysis of BL21 (DE3) ∆*csgBAC* (**B**) and BL21 (DE3)_WT_ (**C**) after cultivation on YESCA plates at 25 °C for 3 d showed that abundant curli fibers were synthesized by BL21 (DE3)_WT_, whereas no fibers were observed on BL21 (DE3) ∆*csgBAC*, indicating that the latter was indeed a curli-deficient mutant. The scale bars represent 1 µm.

Previous studies have demonstrated that *E.coli* curli fibers are only expressed under stressful environmental conditions such as low temperature (below 30 °C), low osmolality, and some other environmental cues (Nutrients, oxygen tension, pH)^17, 28, 29^. Moreover, cells grown on agar plates (YESCA or CFA) produced more curli fibers than cells grown on Liquid medium (YESCA or CFA)^30^. In our TEM analysis, an abundant curli fibers were observed on the surface of *E.coli* grown on YESCA agar plates while no fibers were observed on the surface of *E.coli* grown on YESCA liquid medium. CsgA and CsgB are two related amyloidogenic proteins of curli fibers^21^. In previous studies, no curli fibers could be detected on deletion of CsgA and CsgB^31^. Here, we knocked out three tandem genes in the *csgBAC* operon (*csgA*, *csgB* and *csgC*) by red homologous recombination to construct the curli-deficient mutant strain *E. coli* BL21 (DE3) ∆*csgBAC* ^32^. A standard amyloid-staining colorimetric dye, CR was used to determine curli production in the wild-type and mutant strains^33^. Strains BL21 (DE3) and BL21 (DE3) ∆*csgBAC* were grown on YESCA-CR plates at 25 °C. After 72 h of growth, BL21 (DE3) stained as CR-positive (bright red), whereas BL21 (DE3) ∆*csgBAC* was CR-negative (pale) (Fig. 3A). Furthermore, TEM analysis showed that no fibers were observed on the surface of mutant cells (Fig. 3B) whereas fibers were abundantly produced by the wild-type bacteria (Fig. 3C). Interestingly, the TEM images showed that these fibers were produced in the form of aggregates at the local surface (one end or the other end) of *E.coli*. These data indicate that BL21 (DE3) ∆*csgBAC* lost the ability to synthesize curli fibers and it could be an ideal host for producing extracellular fusions.

### Optimization analysis and membrane localization of CsgG

**Figure 4:**
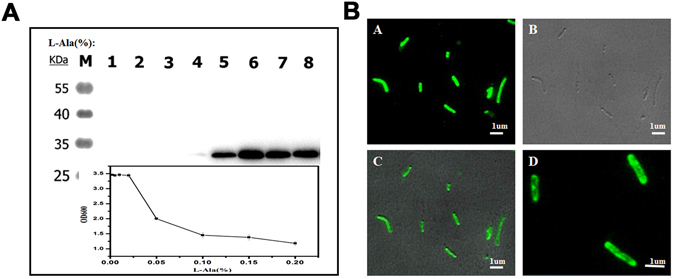
Overexpression optimization and Fluorescence images of CsgG in BL21 (DE3) ∆*csgBAC*. (**A**): Overexpression optimization of CsgG; as shown in the lower part of the chart, cell growth was dramatically inhibited by increasing concentrations of L-Ala. Hence, an optimization experiment for CsgG overexpression was conducted. Blots were probed with anti-His. Lanes 1 to 8 represent induction with the following concentrations of L-Ala (w/v): 0, 0.002, 0.005, 0.01, 0.02, 0.05, 0.1, and 0.15 (%). A concentration of 0.05% L-Ala was found to be optimal to facilitate CsgG expression while allowing cell growth. (**B**): Fluorescence images of CsgG in BL21 (DE3) ∆*csgBAC*; Cells containing CsgG-GFP fusions overexpressed in BL21 (DE3) ∆*csgBAC* at 37 °C. Images show cells examined after the induction of fusion protein synthesis for 3 h. The fluorescent and bright image are shown individually in a and b; c indicates the merge image of a and b; the partial magnification of a is shown in d. The scale bars represent 1 µm.

In previous studies, it was concluded that CsgG overexpression is able to permeabilize the OM, which renders *E. coli* sensitive to erythromycin^31, 34^. Goal’s structural data implied that CsgG could form an ungated peptide diffusion channel in the OM^34^. We introduced a plasmid (pETG) which directs CsgG overproduction inducible by L-Ala into mutant BL21 (DE3) ∆*csgBAC*. Unfortunately, cell growth was obviously inhibited by induction with L-Ala. This implied that overexpression of CsgG may be toxic to the cells. Analysis of published data suggested that excessive amounts of CsgG may form pores in the OM which disrupt the balance of osmotic pressure across the membrane and eventually lead to cell death^20^. Therefore, *csgG* expression was optimized; induction with 0.05% L-Ala proved to be optimal (Fig. 4A).

Mutagenesis of CsgG indicated that its activity is dependent on localization to the OM^34^. To investigate whether the overexpressed CsgG could successfully locate to the OM here, fluorescence microscopy of CsgG-GFP fusions was conducted. A plasmid encoding a CsgG-GFP fusion was transformed into BL21 (DE3) ∆*csgBAC* cells and expressed in cells cultured in LB liquid broth. After 3 h at 37 °C, the cells were examined by fluorescence microscopy (Fig. 4B). As a hydrophobin, CsgG tends to aggregate and eventually form aggregates inside cells if it cannot successfully locate in the OM. As Fig. 4B shows, most cells with expressed CsgG-GFP fusions exhibited diffuse fluorescence and no brilliant fluorescent foci were observed; thus, plasmid encoded CsgG successfully located on the OM judged by criteria from other reports^35^. It was also found that there were several cells exhibiting brilliant fluorescent foci (Fig. 4B–d). This indicates that only a certain amount of CsgG can locate in the OM and allow normal cell growth without leading to cell death, which is consistent with the findings of our experiments on optimization of CsgG expression (Fig. 4A).

### RFP fusion protein can successfully form extracellular amyloid aggregates

**Figure 5:**
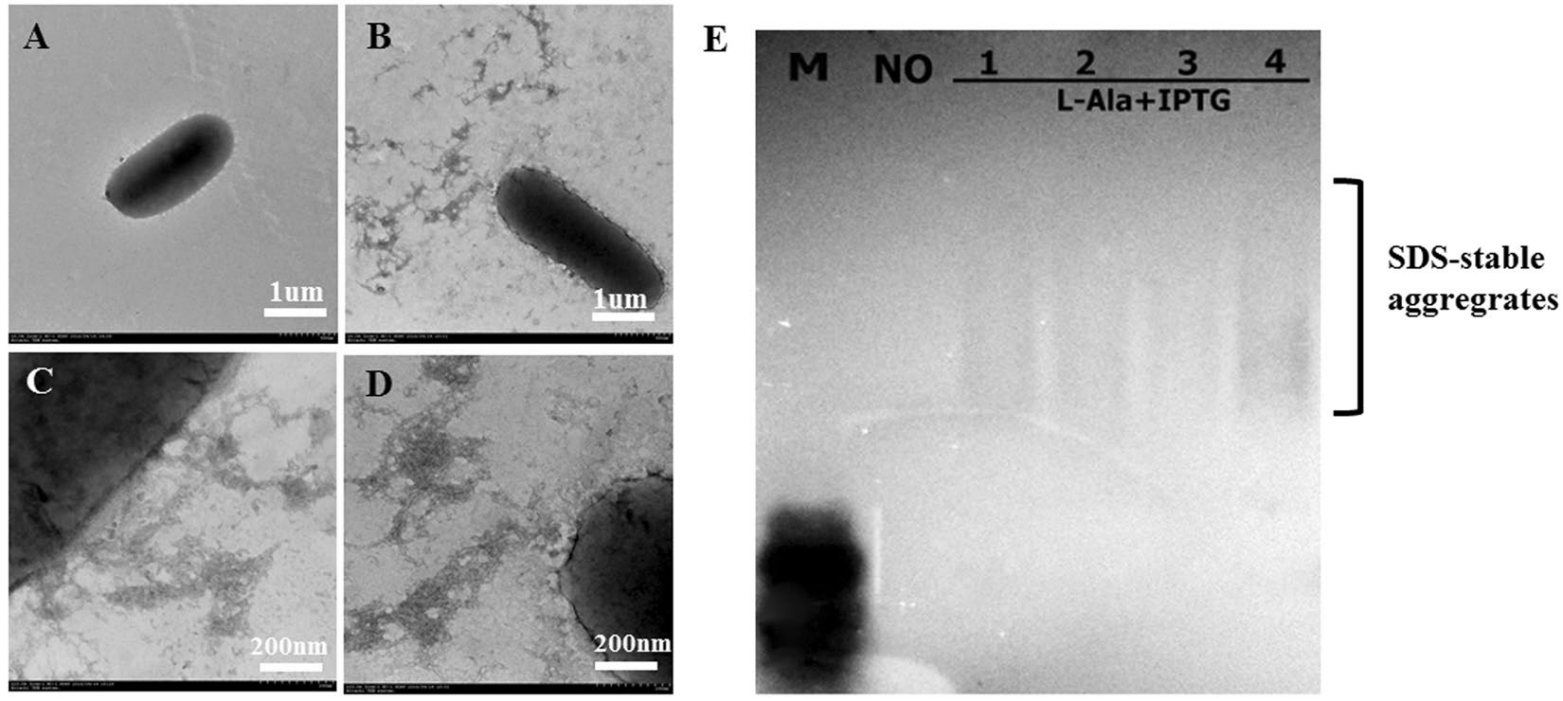
Transmission electron microscopy (TEM) analysis and semi-denaturing detergent agarose gel electrophoresis (SDD-AGE) detection of RFP-M-sup35NM fusion protein. Cells transformed with a plasmid encoding RFP-M-sup35NM fusions were cultured on YESCA uninduced plates (**A**) or induced plates (**B**,**C**,**D**) at 25 °C for 3 d. Samples were also analyzed by SDD-AGE, “No” indicates that cells were not induced with IPTG; lanes 1 to 4 represent four induced samples.

We further investigated whether C-M-sup35NM fusions can be expressed and secreted through the CsgG diffusion channel. In previous studies, prion sup35NM could successfully form amyloid aggregates, which relied on the Csg secretion system of *E. coli* cells^21^. We constructed a RFP-M-sup35NM fusion protein in which we replaced cecropin A with RFP and verified it by fluorescence microscopy. A plasmid encoding RFP-M-sup35NM with a signal peptide (CsgAss) was introduced into BL21 (DE3) ∆*csgBAC* and the RFP-M-sup35NM fusion and CsgG + GFP were co-expressed in mutant *E. coli*. We plated the cells onto YESCA inducing medium (Kan + L-Ala + IPTG) at 25 °C for 3 d. As shown in Fig. S1A–C, brilliant green and red fluorescence were detected, which indicated that CsgG and RFP-M-sup35 fusions were successfully expressed. TEM analysis was also conducted to verify whether the RFP-M-sup35 fusions could be successfully secreted to the cell surface of BL21 (DE3) ∆*csgBAC*. Cells producing CsgG and RFP-M-sup35NM fusions were plated on inducing medium (Kan + L-Ala + IPTG), and non-inducing medium (Kan) was used as a control. An abundance of fibrillar aggregates was detected on the cells grown on inducing medium (Fig. 5B–D), but no aggregates were observed in the case of non-inducing medium (Fig. 5A).

To determine whether those observed fibrillar aggregates (the aggregation state of RFP-M-sup35NM fusions) are in an amyloid-like conformation, we analyzed the fibrillar aggregates using SDD-AGE, which permits the visualization of SDS-stable amyloid polymers^36–38^. Consistent with previous observations, a high-molecular weight smear characteristic of Sup35-derived amyloids was detected for the fibrillar aggregates from cells grown in inducing medium; in contrast, we detected only soluble protein when we examined the fibrillar aggregates from cells cultured in non-inducing medium (Fig. 5E). In summary, RFP-M-sup35NM fusions can be successfully secreted and form amyloid aggregates at the cell surface of BL21 (DE3) ∆c*sgBAC* overexpressing CsgG.

### Determination of extracellular *E. coli* cecropin A fusions

**Figure 6:**
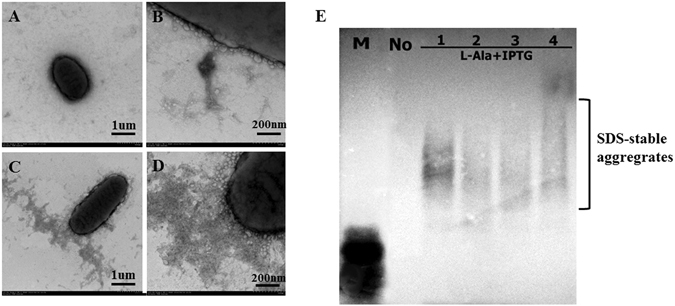
TEM analysis and SDD-AGE detection of C-M-sup35NM fusion protein. Cells transformed with plasmid encoding C-M-sup35NM fusions were cultured on YESCA uninduced plates (**A**) or induced plates (**B**,**C**,**D**) at 25 °C for 3 d. Samples were also analyzed by SDD-AGE. “No” indicates that cells were not induced with IPTG; lanes 1 to 4 represent four induced samples.

The experiments above confirmed that it is practical and feasible to use cells to export RFP-M-sup35NM fusions. We next investigated whether C-M-sup35NM fusions could be secreted and form amyloid aggregates. C-M-sup35NM transformants were cultivated and expressed on YESCA inducing plates containing 0.05% L-Ala and 1 mM IPTG. As a control, non-inducing YESCA plates were used. After 3 d at 25 °C, the cells were scraped, resuspended in sterilized PBS and examined by TEM. Cells from inducing plates revealed an abundance of fibrillar aggregates (Fig. 6B–D), while no such aggregates were visible on cells from non-inducing plates (Fig. 6A). SDD-AGE analysis also indicated that the aggregates from inducing plated were amyloid-like. Consistent with our previous results, fusion protein C-M-sup35NM could also be secreted as amyloid aggregates on the cell surface of *E. coli* BL21 (DE3) ∆*csgBAC* via the curli system (Fig. 6E).

### Release and concentration of cecropin A from extracellular amyloid aggregates

*Mxe* GyrA intein, a natural mini intein, lacks a central intein endonuclease domain; cleavage is activated by the addition of a thiol nucleophile such as DTT, and the target protein is released from the N-terminus of the *Mxe* GyrA intein^22, 39^. A cleavage efficiency of *Mxe* GyrA intein with about 55.2%-72.4% were calculated in some reports^40, 41^, which is consistent with our pre-experiments. Here, cells transformed with plasmid pExport1 and pETG (encoding C-M-sup35NM fusion and CsgG respectively) were cultured on YESCA inducing plates at 25° for 3 d, and then cells that had produced the amyloid aggregates were harvested by scraping, resuspended in PBS and centrifuged at 7500 g for 20 min. The pellets were collected and washed twice with buffer S1. Then the pellets were resuspended in the same volume of buffer S2.

**Figure 7:**
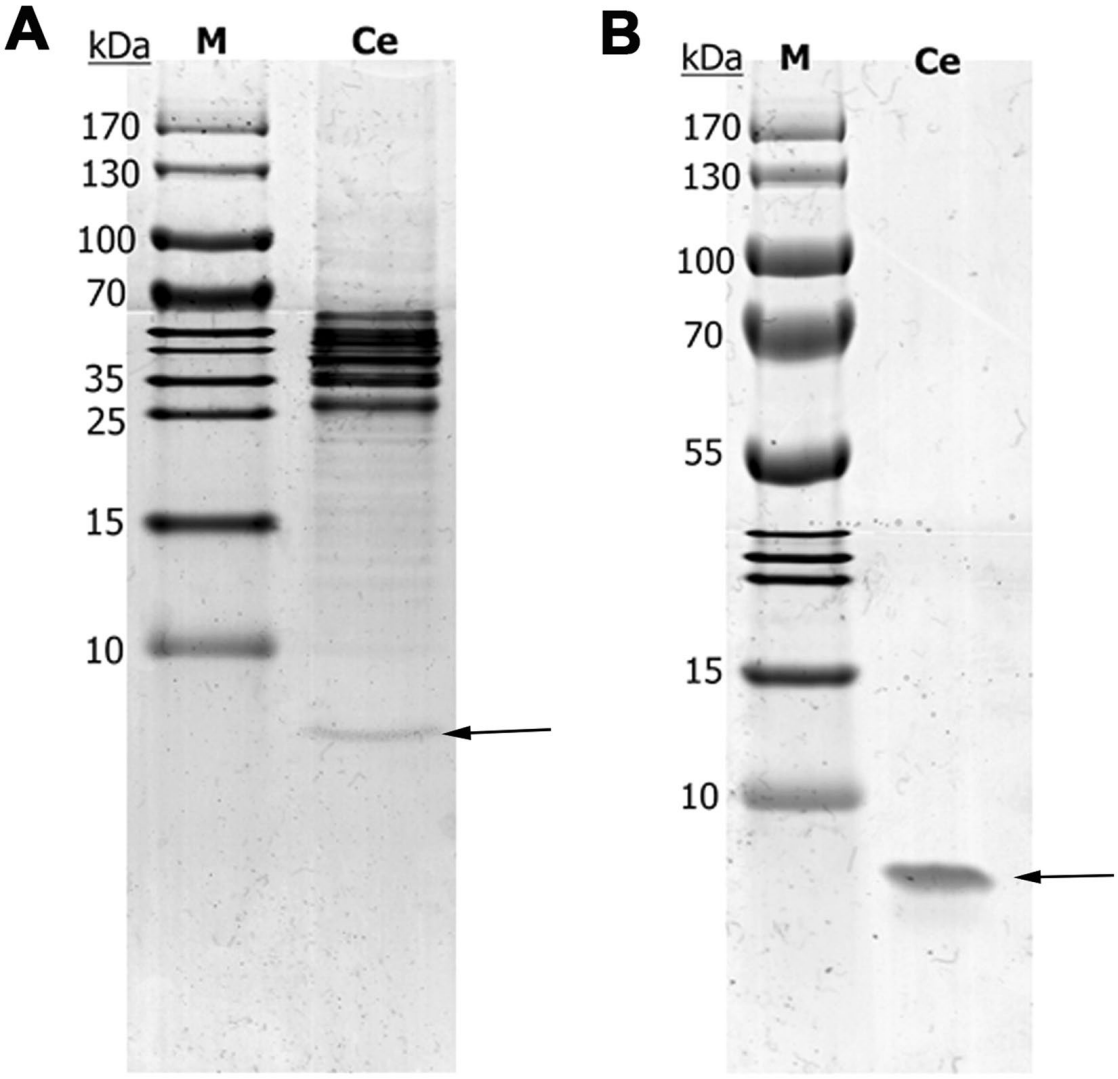
Tricine-SDS-PAGE analysis of cecropin A peptide after cleavage and purification. (**A**) The high gray-level resolution of tricine-SDS-PAGE cropped from Supplementary Figure S2 of the supernatant after cleavage at 4 °C for 12 h; (**B**) Tricine-SDS-PAGE cropped from Supplementary Figure S3 of the purified and concentrated cecropin A peptide; The cecropin A peptides are marked by arrows; M: protein molecular weight ladder.

To obtain the highest yield of cecropin A peptide, different cleavage conditions were tested (4 °C, 12 h/24 h, pH 8.5). Incubation of the samples for 12 and 24 h generated a similar amount of soluble cecropin A peptide in the supernatant after centrifugation at 11,000 rpm for 30 min. However, there were more impurities in the supernatant of samples incubated for 24 h than for 12 h. We performed all subsequent cleavage reactions at 4 °C for 12 h. unfortunately, impurities were also present in the supernatant after cleavage in these conditions (Fig. 7A). We speculated that the toxicity of DTT to cell membranes may be the main reason and that it caused cell lysis and release of intracellular substances. To improve the purity of the cecropin A peptide, a simple ultrafiltration (3 kDa ultrafiltration tube) at 4500 g was applied. As shown in Fig. 7B, the impurities disappeared after ultrafiltration and concentration, and the purity of the final cecropin A peptides was up to 95%. The concentration was determined to be 294 ng/µl using a BCA Protein Assay Kit.

### Determination of the MIC of cecropin A

Over the past two decades, many researchers have focused on how the AMP cecropin A kills bacteria. Cecropin A is a linear, cationic, 37-residue peptide which has a strong interaction with, and forms an amphipathic α-helix, upon binding to membranes^42^. To investigate the effect of cecropin A on membranes, Axelsen’s lab examined the ability of cecropin A to alter membrane permeability in Gram-negative bacteria, which indicated that the effect of cecropin A is concentration dependent and that it kills bacteria by dissipating transmembrane electrochemical ion gradients^43, 44^. They also examined the transcription profiles in *E. coli* treated with sublethal and lethal concentrations of cecropin A and found that expression of 26 genes changed significantly, which demonstrated that cecropin A could induce a genomic response in *E. coli* apart from any lethal effects on the membrane^45^. However, the precise mechanism by which cecropin A affects bacteria remains elusive.

**Figure 8:**
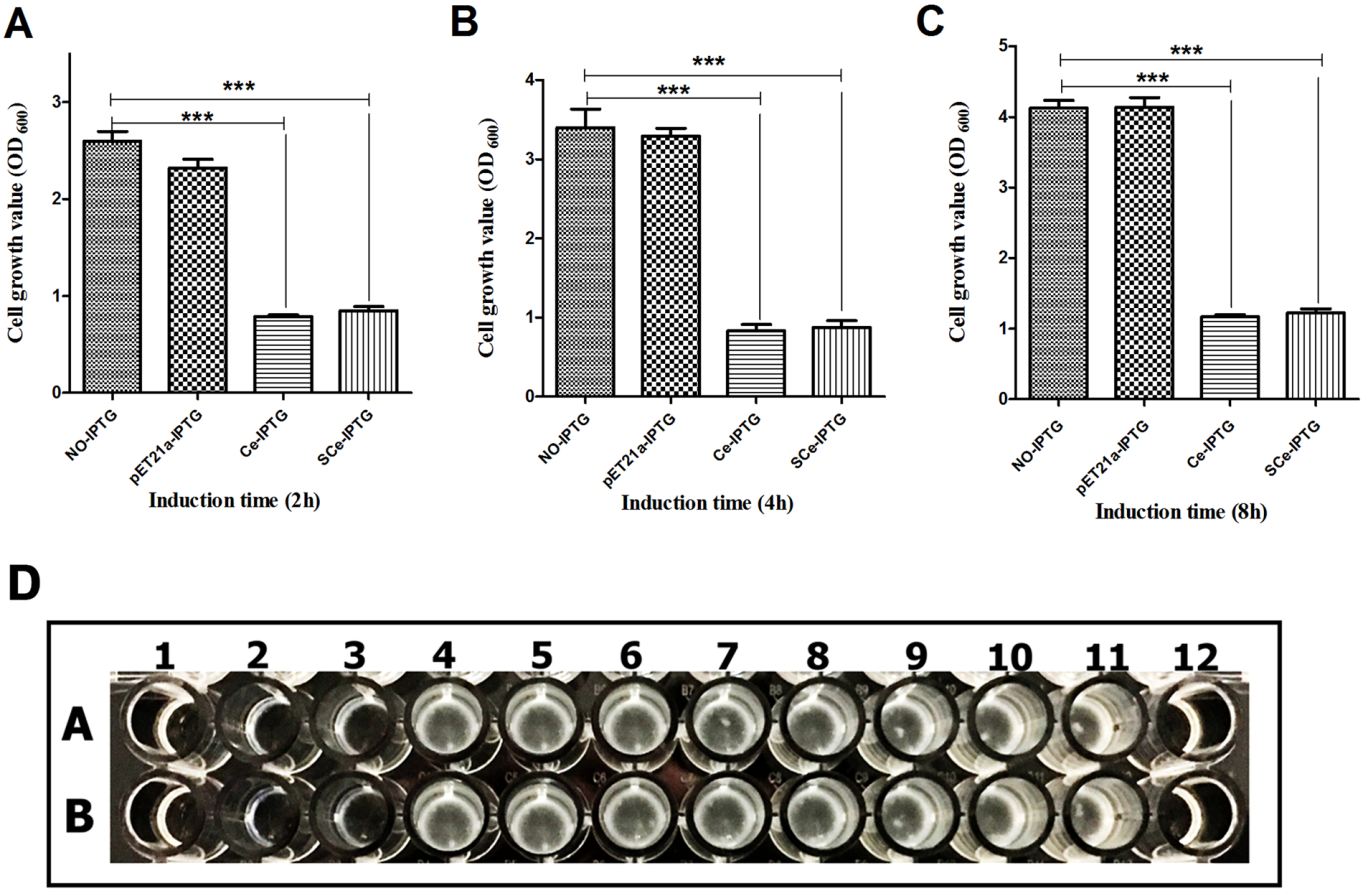
Growth of cells expressing Ce and SCe and inhibition concentration tests of *E. coli ATCC 25922*. (**A**–**C**): Growth of cells expressing Ce and SCe; *E. coli* BL21 (DE3) cells harboring plasmids encoding peptides Ce or SCe were cultured at 37 °C, and cells were induced with 1 mM IPTG when OD_600_ reached 0.8. Cell growth was monitored by OD_600_. The y-axis indicates the OD_600_; the x-axis indicates the time after the cells were induced with IPTG. Ce: Cecropin A peptide; SCe: cecropin A peptide with the extra N-terminal amino acid residues; No-IPTG: cells were not induced with IPTG; pET21a-IPTG: cells harboring plasmid pET21a were induced with IPTG (negative control). Ce-IPTG: cells harboring Ce peptide were induced with IPTG; SCe-IPTG: cells harboring the plasmid encoding the SCe peptide were induced with IPTG. ***p < 0.001. (**D**): Inhibition concentration tests of *E. coli ATCC 25922*; a: Bioproduced cecropin A peptide; b: chemically synthesized cecropin A peptide. Wells 1 to 10 represent concentrations of peptide: 280, 140, 70, 35, 17.5, 8.75, 4.375, 2.188, 1.094, and 0.547 ng/µl. 11. Growth control (broth with bacterial inoculum without antibiotic); 12. Sterilized control (broth only).

In our study, the cecropin A peptide released contained extra N-terminal amino acid residues which remained after Sec translocation. To verify whether these additional N-terminal amino acid residues influenced the antibacterial activity of cecropin A, a validation experiment was performed by construction of two plasmids (pET-ce and pET-sce) encoding cecropin A (Ce) or cecropin A with the extra N-terminal amino acid residues (SCe), respectively. *E. coli* BL21 (DE3) cells transformed with pET-ce or pET-sce were cultured and then induced with IPTG for 10 h, and the cell growth was monitored by OD_600_. Cells expressing the SCe peptide were dramatically inhibited when induced with IPTG; around 67.4% of the cells were killed after 2 h, which was consistent with cells expressing Ce (cell mortality rate 69.6%) (Fig. 8A). This indicated that the SCe peptide had the same antibacterial activity as the Ce peptide. Moreover, about 74.2% cells were killed by SCe and 75.6% by Ce after 4-h induction (Fig. 8B). These findings show that the expressed SCe peptide and Ce peptide are stable in the intracellular environment. Cell death did not increase further with elongated induction (8 h; Fig. 8C); the cell death rate was then 70.4% in cells expressing SCe and 71.8% in cells expressing Ce, illustrating that expression of the SCe and Ce peptides reached a plateau.

These results indicated the SCe peptide had the same antibacterial properties as cecropin A peptide, i.e., that the extra N-terminal amino acid residues had no effect on the antibacterial activity of cecropin A. Previous studies have reported that α-helical peptides initially bind to the bacterial membrane, then form water-filled pores to release the cytoplasmic contents and cause cell death^46–48^. In our study, the additional N-terminal amino acid residues may not disrupt the structure of cecropin A, so it can still successfully bind to the membrane and form water-filled pores.

The antimicrobial activity of the bioproduced cecropin A peptide (equivalent to SCe) was determined using MIC tests against *E. coli* ATCC 25922 with chemically synthesized cecropin A used in controls. A 280 ng/µl concentrate of bioproduced cecropin A peptide and synthetic cecropin A peptide were used as the starting concentration for MIC tests, followed by twofold serial dilutions. To ensure the tests were accurate and valid, the final inoculum size for broth dilution was 5 × 10^5^ CFU/ml^49^. As Fig. 8D shows, the bioproduced cecropin A had the same antimicrobial activity as synthetic cecropin A, indicating that our strategy for producing cecropin A was effective and feasible.

## Conclusion

Many attempts have been made in the production of AMPs over past decades but with little success. In this work, a novel extracellular secretion expression and online-cleavage strategy is shown, which indicates that AMPs can be produced by a simple and low-cost process. Based on the curli system of *E. coli*, a tandem fusion of an AMP (cecropin A), a self-cleavage intein (*Mxe* GyrA), and a yeast prion protein (sup35NM) was designed. The cecropin A fusion protein was expressed and secreted to the cell surface of *E. coli* in the form of insoluble extracellular amyloid aggregates. The fusions could be collected by simple centrifugation, eliminating tedious cell disruption and expensive chromatography-based purification steps. Finally, cecropin A peptide was released by addition of DTT. MIC results demonstrated that the bioproduced peptide cecropin A had the same antimicrobial properties as chemically synthesized cecropin A. Our study shows a novel strategy and platform for large-scale production of AMPs and other commercially-viable peptides.

## Methods

### Construction of strain and plasmids

The mutant strain *E. coli* BL21 (DE3) ∆*csgBAC* was constructed by replacing the *csgBAC* genes of strain BL21 (DE3) with a kanamycin resistance gene using the lambda Red recombinase system^50^. The *csgG* gene was isolated from *E. coli* K-12 genomic DNA and cloned into expression vector pKJE7 under the control of the arabinose-inducible P_BAD_ promoter. A green fluorescent protein (GFP) reporter gene fused to *csgG* was obtained by overlap extension PCR and cloned into pKJE7, resulting in plasmid pKJE7-csgG-GFP. The nucleotide sequences encoding C-M-sup35NM export-directed fusion proteins contained a signal peptide (csgAss: which is derived from the N-terminal 42 residues of csgA, consisting of a SecA-dependent secretion signal and the CsgG targeting sequence) at the N-terminus, the peptide cecropin A, a self-cleavage intein *Mxe* GyrA, a spacer, sup35NM, and a His_6_-tag at the C terminus; this construct was cloned into pET21a under the control of the IPTG-inducible T7 promoter. Plasmid pET-csgAss-RFP-Mxe-sup35NM was obtained by replacing the cecropin A gene with a red fluorescent protein (RFP) gene. All clones were verified by restriction enzyme mapping and DNA sequencing. Strains, plasmids and primers used in this study are described in Supplementary Tables S1 and S2.

### Cell growth and curli biofilm formation

*E. coli* strains DH5α and BL21 (DE3) were grown in Luria Bertani (LB) media supplemented with appropriate antibiotics (100 µg/ml ampicillin, 50 µg/ml kanamycin or 25 µg/ml chloramphenicol) at 37 °C for gene manipulation. To produce curli, the wild-type BL21 (DE3) cells or mutant BL21 (DE3) ∆*csgBAC* transformed with compatible plasmids directing the synthesis of CsgG and the C-M-sup35NM export-directed fusion protein were cultured overnight. Then, 1 ml of the broth was diluted to OD_600 nm_ = 0.01 in LB supplemented with appropriate antibiotics (ampicillin and chloramphenicol) and cultured at 37 °C for 30 min. Ten microliters of the culture were streaked or spotted onto YESCA plates^14^ containing 10 g L^−1^ of casamino acids, 1 g L^−1^ of yeast extract and 20 g L^−1^ of agar. The YESCA plates were supplemented with the appropriate inducers (0.05% [w/v] L-arabinose, 1 mM IPTG) and antibiotics. Plates were then incubated at 25 °C for 3 d.

### Congo red (CR) binding experiment

Overnight cultures of the wild-type BL21 (DE3) and mutant strain BL21 (DE3) ∆*csgBAC* cells at 37 °C were spotted onto YESCA-CR plates (YESCA plates + 50 µg/ml CR) supplemented with the appropriate inducers and antibiotics as described above. The plates were incubated at 25 °C for 3 d and then imaged to determine the extent of CR binding.

### Transmission electron microscopy (TEM) and fluorescence microscopy

Cell samples, either taken directly from induced YESCA liquid cultures or scraped from YESCA plates, were adsorbed onto carbon or formvar/carbon-coated nickel grids in sterilized PBS and air dried for 2–5 min. The cells were washed by floating the grid on 20 µl of distilled water and blotted dry; next, cells were negatively stained with 3% phosphotungstic acid for 2 min and blotted dry again. The washing procedure in distilled water was repeated twice, and then the cells were viewed on a Hitachi H-7650 (Japan) transmission electron microscope. For fluorescence microscopy, cells were resuspended in sterilized PBS (1% w/v) and spotted onto a slide, air dried, then visualized with a 63 × phase contrast objective on a Leica DMI 6000 microscope.

### Semi-denaturing detergent agarose gel electrophoresis (SDD-AGE)

SDD-AGE was conducted according to the method published by Halfmann and Lindquist^51^. Amyloid proteins were separated by 1.5% agarose gel electrophoresis. Blots were probed with anti-His (Abcam), detected using an ECL plus Western Blotting Detection System (GE Healthcare).

### Collection and concentration of cecropin A by intein-mediated cleavage

*E. coli* BL21 (DE3) ∆*csgBAC* harboring the export-directed plasmid were spread onto YESCA plates supplemented with 100 µg/ml ampicillin, 50 µg/ml kanamycin, 25 µg/ml chloramphenicol and 1 mM IPTG. After cultivation at 25 °C for 3 d, the cells were harvested from the plates and resuspended in buffer S1 (20 mM Tris-HCl, 500 mM NaCl, 1 mM disodium edetate (EDTA), pH 8.5) to 10 OD_600_ culture/ml, followed by centrifugation at 7500 g for 15 min at 4 °C. The precipitates were washed twice with buffer S1, and resuspended in the same volume of buffer S2 (20 mM Tris-HCl, 500 mM NaCl, 1 mM EDTA and 40 mM dithiothreitol (DTT), pH 8.5) to start cleavage at 4 °C for 12 or 24 h. Centrifugation at 11,000 rpm for 30 min was applied, and the supernatant was further purified by ultrafiltration and concentrated by PEG20000. To remove DTT, concentrated supernatant was dialyzed into buffer S3 (20 mM Tris-HCl, 50 mM NaCl, pH 8.5).

### Minimal inhibitory concentration (MIC) test

The MIC of the bioproduced cecropin A against bacteria was measured by a broth microdilution method as previously reported^49^; *E. coli* ATCC 25922 was used to quantify the antimicrobial activity of the peptide. Overnight cultures of *E. coli* were diluted with fresh Mueller-Hinton Broth (MHB) to OD 0.08–0.13 at 625 nm (0.5 McFarland standards)^49^, and then further diluted 1:100 with fresh MHB; the final concentration was approximately 10^7^ colony-forming units (CFU) per ml. The bioproduced cecropin A was serially diluted from 280 mg/ml into MHB medium and 50 µl aliquots of this diluted cecropin A were added into 96-well plates. Subsequently, standardized bacterial suspension (50 µl) was added to each well. Growth controls (broth with bacterial inoculum without antibiotics) contained no peptide antibiotic, and sterility controls contained only broth. The plates were incubated at 37 °C for 16 h, and the lowest concentration of the peptide at which the growth of *E. coli* was prevented (as indicated by the lack of visible turbidity) was recorded. Growth controls demonstrated visible turbidity, while the sterile controls remained clear.

## Electronic supplementary material

Supplementary Information
