# Supplementary material for: A novel secretion and online-cleavage strategy for production of cecropin A in Escherichia coli
Source: Sci Rep. 2017 Aug 4;7:7368. doi: 10.1038/s41598-017-07411-5 (PMC5544755; doi:10.1038/s41598-017-07411-5)
Supplement: Supplementary file 1 — Supplementary Information [file 41598_2017_7411_MOESM1_ESM.docx]

**A novel secretion and online-cleavage strategy for production of cecropin A in *Escherichia coli***

Meng Wang^1^, Minhua Huang^1^, Junjie Zhang^1^, Yi Ma^1^, Shan Li^1^, Jufang Wang^1, 2*^

^1^School of Bioscience and Bioengineering, South China University of Technology, Guangzhou 510006, China

^2^Guangdong Key Laboratory of Fermentation and Enzyme Engineering, School of Bioscience and Bioengineering, South China University of Technology, Guangzhou 510006, China; telephone: 86-20-39380626, Fax: 86-20-39380626, e-mail: jufwang@scut.edu.cn

**Fig. S1 Fluorescence images of CsgG and RFP-M-sup35NM fusion protein in *E. coli* BL21 (DE3) ∆*csgBAC***

Green fluorescence image of CsgG (A); Red fluorescence image of RFP-M-sup35 fusion (B); Overlay fluorescence image of a field of cells containing CsgG and RFP-M-sup35 fusion proteins (C).

**Fig. S2 Full-length tricine-SDS-PAGE of the supernatant after cleavage at 4°C for 12 h**

Ce: the supernatant after cleavage at 4°C for 12 h, corresponding cecropin A peptide (cleaved from fusions) position is marked by arrow. M: protein molecular weight ladder.

**Fig. S3 Full-length tricine-SDS-PAGE of t****he purified and concentrated cecropin A peptide**

Ce: the purified and concentrated cecropin A peptide (marked by arrow); M: protein molecular weight ladder.

**Table S1 Plasmids and strains used in this study**

**Table S2 Primers used in this study**

**
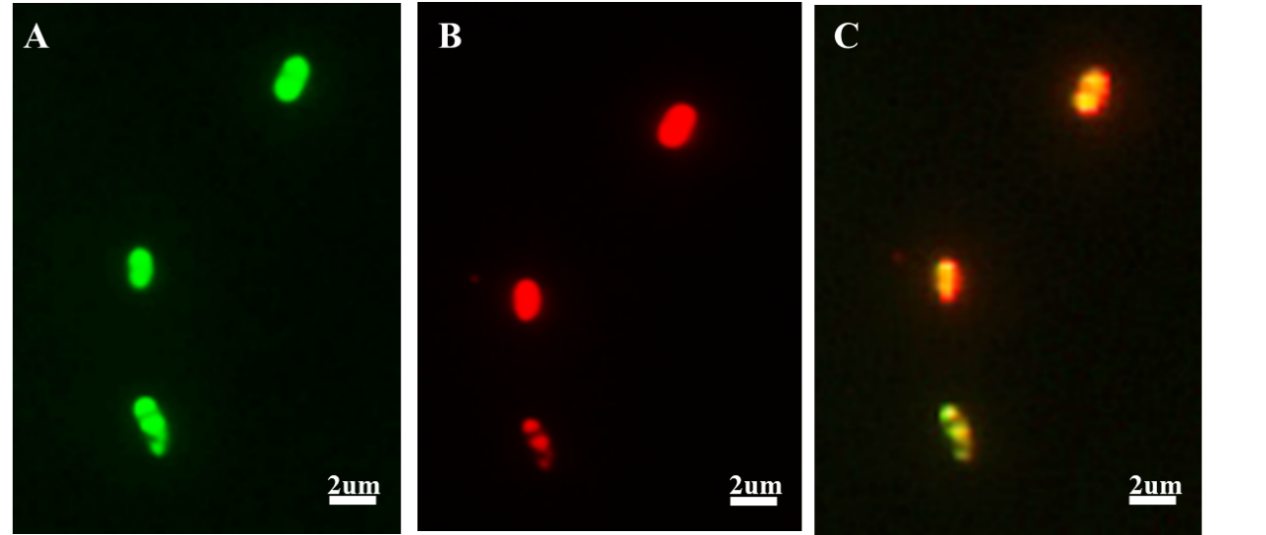
**

Figure S1

**
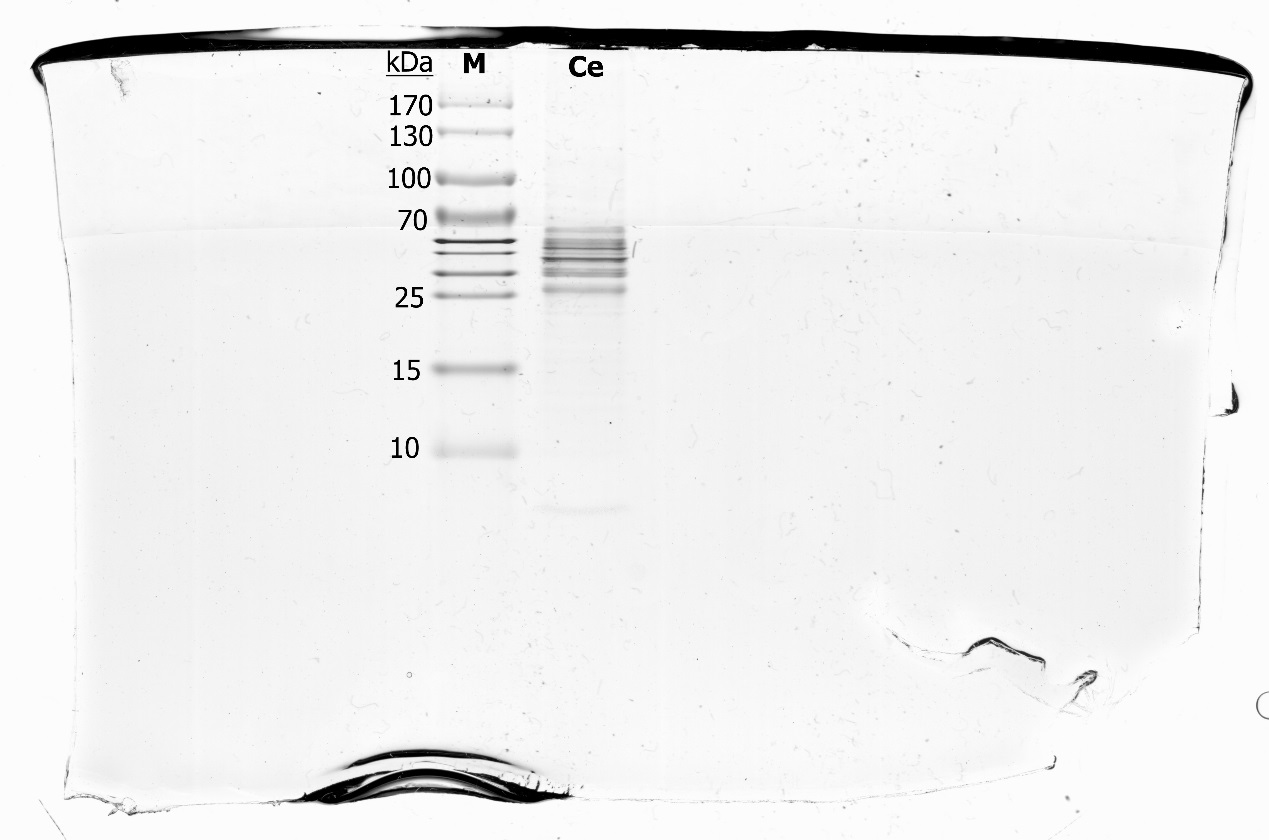
**

**Fig. S2**

**
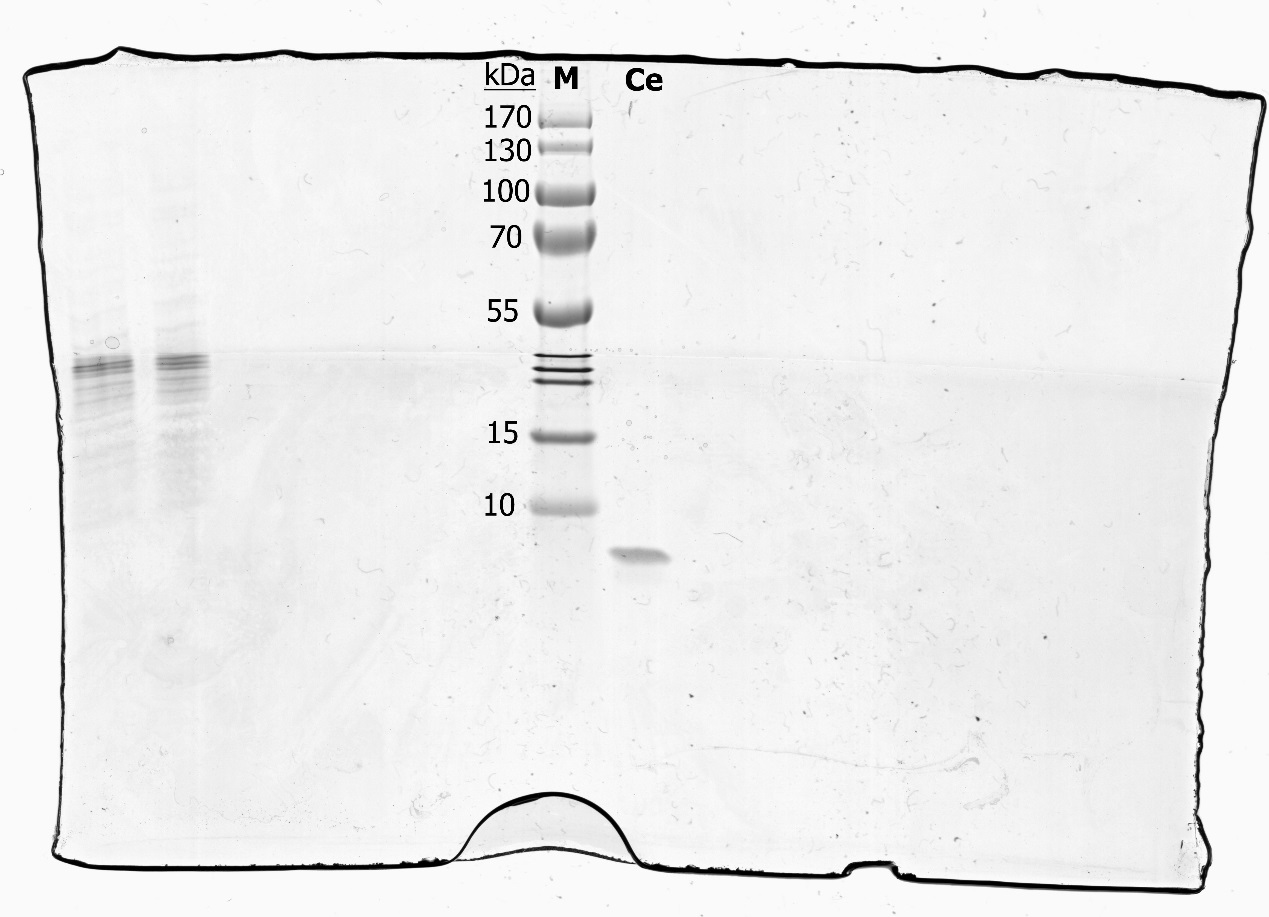
**

**Fig. S3**

**Table S1 Plasmids and strains used in this study**

| **Strain/plasmid** | **Genotype or relevant characteristics** | **Source/Ref** |
| --- | --- | --- |
|  |  |  |
| **Strain** |  |  |
| DH5α | F– Φ80lacZΔM15 Δ(lacZYA-argF) U169 recA1 endA1 hsdR17 (rK–, mK+) phoA supE44 λ– thi-1 gyrA96 relA1 | Taraka(Da Lian) |
| BL21(DE3) | F– ompT gal dcm lon hsdSB(rB- mB-) λ(DE3 [lacI lacUV5-T7 gene 1 ind1 sam7 nin5]) | Taraka(Da Lian) |
| BL21(DE3)△csgBAC | BL21(DE3)*Δ(csgBAC)(::kanR)* | This study |
|  |  |  |
| **Plasmid** |  |  |
| pKJE7 | *Bla* PBAD *dnaK-dnaJ-grpE; pACYC ori;* | This study |
| pET21a | *T7 promotor; pBR322 ori;* | This study |
| pKJE7-csgG | *Bla* PBAD *csg-His_6_; pBR322 ori;* *produces CsgG fused with His_6_* | This study |
| pKJE7-csgG-GFP | *Bla* PBAD *csg-GFP; pBR322 ori;* *produces CsgG fused with GFP* | This study |
| pET-csgAss-RFP-Mxe-sup35 | *T7* *csgAss-RFP-Mxe-sup35-His_6_; pBR322 ori;* *produces Fusion protein:* *csgAss-RFP-Mxe-sup35-His_6_* | This study |
| pET-csgAss-Ceropin-Mxe-sup35 | *T7* *csgAss-Ceropin-Mxe-sup35-His_6_; pBR322 ori;* *produces Fusion protein:* *csgAss-Ceropin-Mxe-sup35-His_6_* | This study |
| pET-ce | *T7 cecripin; pBR322 ori;* *produces ceceopin A peptide(Ce)* | This study |
| pET-sce | *T7 Sig-cecropin*; *pBR322 ori;* *produces cecropin A peptide with N terminal amino acids(SCe)* | This study |

**Table S2 Primers used in this study**

**
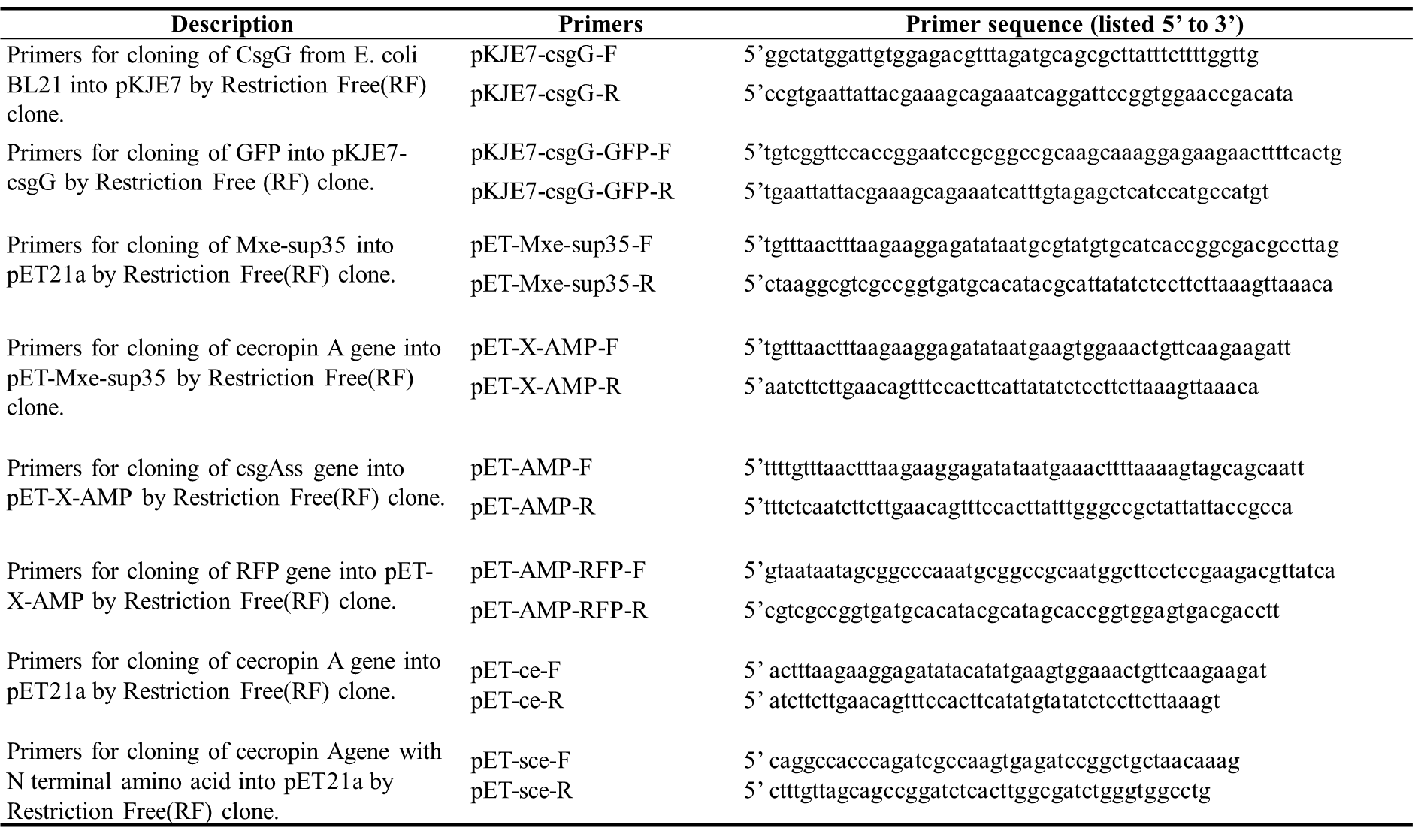
**
